# Supplementary material for: The Laro Kwo Project: A train the trainer model combined with mobile health technology for community health workers in Northern Uganda
Source: PLOS Glob Public Health. 2023 May 17;3(5):e0001290. doi: 10.1371/journal.pgph.0001290 (PMC10191267; doi:10.1371/journal.pgph.0001290)
Supplement: Attachment — Submitted filename: REVISION Ebbs Plos GPH Cover .docx [file pgph.0001290.s002.docx]

Yale School of Medicine

*Section of Critical Care Medicine*

*Department of Pediatrics*

*Department of Pediatrics*

February 14, 2023

Dr. Roopa Shivashankar

Academic Editor

And

Editorial Staff

*PLoS Global Public Health*

*Re: Response to Reviews*

The authors of this manuscript would like to thank the reviewers and editors of Plos Global Public Health for their time invested in reviewing our submission. We found the comments extremely beneficial in the improvement of this manuscript. Per journal requirements, we have submitted the inclusivity in global research questionnaire. We have also uploaded our raw data through *Open Science Framework,* per journal recommendations <https://osf.io/mhd94/?view_only=6fffe121df6a4172a12cc0d17f0f2b64>. Finally, the authors have declared that no competing interests exist. After careful review, we addressed and responded to the following reviewer comments:

***Comments from Reviewer #1:***

*1. The study is well articulated and presents the results comprehensively. The problem statement is clear and the research gap is identified.
2. The involvement of local elected representatives and local village leaders along with other officials for the conceptualisation of LKP and training is a refreshing strategy and a replicable example of communitization.
3. The introduction needs to give context of the Ugandan CHW program. Findings of desk review of CHW policy would be helpful to build the context for the study site.*

Thank you for these comments, we added context and background to CHW training in Uganda (please see lines 70-79)

*4. A flow chart of the rollout of the intervention may be added for showcasing “Train-the-trainer” model adapted by the study. This will also clarify number of participants in the study. Exclusion and inclusion criteria (if any) may also be added.*

Thank you for this comment, we added lines 127-135 that discusses inclusion and exclusion criterion.

*5. The study may also need to include participant profile – age, gender, education qualification, experience in years/ months working as CHW, households/ population covered (if applicable) etc. Adding these details shall be helpful for understanding the study setting. Additionally, a baseline data of CHW/participant’s digital literacy could be mentioned, if gathered. This would also set a context of a pre-training for use of mobile applications.*

Thank you for these comments, we agree that this is important and added lines 123-135 in addition to Table 1 to highlight CHW characteristics and basic demographics. We also added exposure to digital literacy as suggested.

*6. The study does not mention the background qualification/skill set of the CHWs which could be an important confounding factor in assessing the retention of the training. Any other perceived confounding factors could also be mentioned.*

Thank you for this comment; in addition to above, we added a section on confounding variables and bias on lines 157-161.

*7. Limitations of the study need to be mentioned. A section on scope for supplementing the initiative with further research could be mentioned.*

Thank you, we agree with this comment and added lines 215-223 to address limitations of this study in addition to lines 245-248 addressing our initiatives for further/future research.

*8. The study does not mention whether it has adhered to STORBE guidelines. The study may reframe if it is deemed fit by the journal. (appraisal against STROBE checklist is attached as Annexure)*

Thank you for this comment and the appraisal against STROBE checklist; this is extremely helpful and we responded to the missing components to the checklist as annotated:

Item 5: addressed above (#5), setting described in more detail in addition to CHW characteristics and study period.

Item 6: addressed above, (#4-6)

Item 7: Confounders addressed above (#6)

Item 9: Selection bias included and more details on inclusions/exclusion were added as addressed above (#6)

Item 10: Sample Size: We included lines 163-166, rationale for our sample size.

Item 12: Statistical methods: Lines 203-205 were added to describe lost to follow-up.

Item 13: Participants: Outlined additional detail on participants on lines 203-205 including lost to follow-up.

Item 14: Lines 203-205 add information on missing data and lost to follow-up. Table 1 was added and highlights additional participant information.

Item 16: We do not believe is applicable to this study.

Item 19: Addressed above, with the addition of lines 215-223.

***Comments from Reviewer #2:***

*1. The structured IMRaD (Introduction, methods, results and discussion) format of abstract was absent in the manuscript.*

Thank you for this comment. We reviewed the IMRaD format and believe the abstract includes each of the categories.

***2. Introduction:***

*-It would be useful if the following information are also provided to clarify the context for better understanding of the global audience:*

*-Is there any specific name of the community health worker in Uganda?*

*-What is the population catered by each CHW in Uganda generally, i.e. is there a population norm for CHW.*

*-Since what time CHWs have been existing in the health system of Uganda?*

Thank you for these comments, we added context and background to CHW training in Uganda (please see lines 70-79)

*-What are the minimum educational qualifications required to be a CHW, as this would determine the level of acceptance of mobile health technology?*

Thank you for these comments, we agree that this is important and added lines 123-135 in addition to Table 1 to highlight CHW characteristics and basic demographics. We also added exposure to digital literacy as suggested.

-*The sentence on line number 66 and 67 needs to be grammatically modified.*

Thank you for this comment. We reviewed for grammatical errors on these lines.

*-What is train the trainers model as the rationale from various studies have mentioned about the utility and the sustainability of the model?*

*-Who were the stakeholders required to run this train the trainer model apart from the community leaders and CHWs as per the literature?*

Thank you for these comments. We added lines 85-89 to expand on the utility and sustainability of this model from past studies, particularly in the context of a limited health resource setting. Further, we added information on stakeholders per the literature.

*-References for the line number 76, 84 and 85 should be provided.*

Thank you for this comment, we added reference citations to these lines.

***3. Methodology:***

*-Study period of the three year training is not mentioned unlike the project (2016)*

Thank you for this comment, we added additional details on project training timeline on lines 119-120 and 127-129.

*-Why exactly 25 CHWs? Is there any specific rationale for including only so much? In other words, how was sample size calculated? Sampling technique should be added.*

Thank you for these comments, we added details re: sample size calculation on lines 167-170 and sampling technique on lines 161-165

*-Who was the instructor for the didactic training among the team? This needs to mentioned.*

*-In evaluating skills for consistency by the instructor, was any skill out of 6 medical skills enlisted in Table 1 used for evaluation or was it a mere repetition of only one consistency skill by all 25?*

Thank you for these comments, we added further details on lines 146-151 to clarify the didactic and evaluation process.

*-How was the evaluation performed through checklist? If it was through a checklist, then was it a validated one? What was the duration of evaluation of each competency?*

Thank you for these comments, we added lines 148-151 to detail validation of competency checklists.

*-Was 25 new CHWs enrolled every year as the initial cohort undergoes second and third year of training leading to 75 CHWs in total? This number 75 has been mentioned only in the acknowledgement. This needs clarification.*

Thank you for this comment, we added additional details to clarify total CHWs enrolled on lines 119-120 and 127-129.

*4. Statistical Analysis:*

*-How data entry was done and its quality was assured, has to be mentioned*

*-Was any software used for data analysis? If yes, then mention it.*

Thank you for these comments, we added lines 191-193 to address data entry and SPS software.

*-Level of statistical significance for P value considered by the author needs to be mentioned.*

Thank you for this comment, we added line 197 to answer this.

*-Was there any data missing? If yes, then mention wherever applicable.*

*-Was there any loss to follow up in the subsequent years?*

Thank you for these comments, we added lines 203-205 to clarify loss to follow-up and missing data.

*5. Results:*

*Descriptive statistics of the cohort including socio-demographic information and years of experience as CHW should be added if available.*

Thank you for this comment, we added lines 123-135 in addition to Table 1 to highlight CHW characteristics and basic demographics.

*The outcome in the objective of comparing the initial trainee scores from the certified health professionals apart from the trained CHWs was not found in the results section.*

Thank you for this comment. Health professionals did not take any competency exams but administered them; the difference is measured between cohorts of CHWs. We hope the above additions to this manuscript clarified this.

*6. Discussion:*

*-Is too short and not all the points have been discussed. This needs to be rewritten.*

*-Limitations and strengths need to mentioned clearly.*

*-Specific recommendations to be given apart from need of future studies.*

Thank you for these comments. We added further details in the discussion which includes limitations and future direction based on our results. Please see lines: 227-235 and 257-260

Thank you very much for your time and consideration,

Sincerely,

Daniel S. Ebbs DO, MS, FAAP

Section of Pediatric Critical Care Medicine

Department of Pediatrics

Yale University and Yale New Haven Hospital

New Haven, Connecticut

[Daniel.Ebbs@yale.edu](mailto:Daniel.Ebbs@yale.edu)
